# A pilot investigation of differential hydroxymethylation levels in patient-derived neural stem cells implicates altered cortical development in bipolar disorder

**DOI:** 10.3389/fpsyt.2023.1077415

**Published:** 2023-04-17

**Authors:** Ashish Kumar, Mark Z. Kos, Donna Roybal, Melanie A. Carless

**Affiliations:** ^1^Department of Cancer Biology, Wake Forest School of Medicine, Winston-Salem, NC, United States; ^2^Population Health Program, Texas Biomedical Research Institute, San Antonio, TX, United States; ^3^South Texas Diabetes and Obesity Institute, Department of Human Genetics, The University of Texas Rio Grande Valley School of Medicine, San Antonio, TX, United States; ^3^Department of Psychiatry, Division of Child and Adolescent Psychiatry, University of Texas Health Science Center at San Antonio, San Antonio, TX, United States; ^4^Traditions Behavioral Health, Larkspur, CA, United States; ^5^Department of Neuroscience, Developmental and Regenerative Biology, The University of Texas at San Antonio, San Antonio, TX, United States; ^6^Brain Health Consortium, The University of Texas at San Antonio, San Antonio, TX, United States

**Keywords:** hydroxymethylation, bipolar disorder, neuronal differentiation, RRHP, reduced representation 5-hydroxymethylcytosine profiling, neural stem cell (NSC), induced pluripotent stem cell (iPSC)

## Abstract

**Introduction:**

Bipolar disorder (BD) is a chronic mental illness characterized by recurrent episodes of mania and depression and associated with social and cognitive disturbances. Environmental factors, such as maternal smoking and childhood trauma, are believed to modulate risk genotypes and contribute to the pathogenesis of BD, suggesting a key role in epigenetic regulation during neurodevelopment. 5-hydroxymethylcytosine (5hmC) is an epigenetic variant of particular interest, as it is highly expressed in the brain and is implicated in neurodevelopment, and psychiatric and neurological disorders.

**Methods:**

Induced pluripotent stem cells (iPSCs) were generated from the white blood cells of two adolescent patients with bipolar disorder and their same-sex age-matched unaffected siblings (*n* = 4). Further, iPSCs were differentiated into neuronal stem cells (NSCs) and characterized for purity using immuno-fluorescence. We used reduced representation hydroxymethylation profiling (RRHP) to perform genome-wide 5hmC profiling of iPSCs and NSCs, to model 5hmC changes during neuronal differentiation and assess their impact on BD risk. Functional annotation and enrichment testing of genes harboring differentiated 5hmC loci were performed with the online tool DAVID.

**Results:**

Approximately 2 million sites were mapped and quantified, with the majority (68.8%) located in genic regions, with elevated 5hmC levels per site observed for 3’ UTRs, exons, and 2-kb shorelines of CpG islands. Paired t-tests of normalized 5hmC counts between iPSC and NSC cell lines revealed global hypo-hydroxymethylation in NSCs and enrichment of differentially hydroxymethylated sites within genes associated with plasma membrane (FDR = 9.1 × 10^−12^) and axon guidance (FDR = 2.1 × 10^−6^), among other neuronal processes. The most significant difference was observed for a transcription factor binding site for the *KCNK9* gene (*p* = 8.8 × 10^−6^), encoding a potassium channel protein involved in neuronal activity and migration. Protein–protein-interaction (PPI) networking showed significant connectivity (*p* = 3.2 × 10^−10^) between proteins encoded by genes harboring highly differentiated 5hmC sites, with genes involved in axon guidance and ion transmembrane transport forming distinct sub-clusters. Comparison of NSCs of BD cases and unaffected siblings revealed additional patterns of differentiation in hydroxymethylation levels, including sites in genes with functions related to synapse formation and regulation, such as *CUX2* (*p* = 2.4 × 10^−5^) and *DOK-7* (*p* = 3.6 × 10^−3^), as well as an enrichment of genes involved in the extracellular matrix (FDR = 1.0 × 10^−8^).

**Discussion:**

Together, these preliminary results lend evidence toward a potential role for 5hmC in both early neuronal differentiation and BD risk, with validation and more comprehensive characterization to be achieved through follow-up study.

## Introduction

Bipolar disorder (BD) is a complex mental illness characterized by recurrent episodes of mania and typically depression and is associated with progressive social and cognitive disturbances. With a lifetime prevalence of about 2.4% for bipolar spectrum disorders (1), the chronic nature of this condition, combined with an increased risk for suicide (2), high socioeconomic burden (3), and low treatment effectiveness (4, 5), make BD a significant public health issue. Understanding the etiology of BD is critical for the development of improved therapeutic approaches, and given its high heritability, estimated through twin studies (65–80%) (6), genetic factors are likely involved. However, common genetic variants identified to date explain only ~20% of the phenotypic variance for BD (7), and it is likely that rare variants, as well as additive genetic and environmental factors, contribute to the high heritability estimates. Several environmental events have been reported to be associated with a higher incidence of BD, including maternal smoking, prenatal infection, chronic stress, and childhood trauma (8). The likely interaction of such environmental factors with BD risk genes suggests a potential role of epigenetic mechanisms in the genetic liability of BD.

Epigenetic studies of BD, and its treatment with mood stabilizers, have largely focused on DNA methylation profiling in the brain or peripheral blood, identifying both global and regional differential methylation patterns in patients with bipolar disorder versus non-patients, and in patients following treatment with mood stabilizers (9–14). Further, the disruption of epigenetic regulation by environmental factors during embryonic development, childhood, and adolescence has long-term consequences on adult phenotypes and is thought to contribute to risk for psychiatric disorders (15–19). Recently, 5-hydroxymethylcytosine (5hmC) has garnered much attention as a potential key player in neurodevelopment and brain-related disorders. 5hmC expression is high in embryonic stem cells, where it contributes to pluripotency, and is diminished upon differentiation, except for mature neuronal cells (20). Fetal brains express high levels of 5hmC, with widespread changes seen throughout human brain development (21). Adult brains show even higher expression, as well as differential expression across neuronal cell types and brain regions (22, 23). 5hmC has therefore been implicated in various psychiatric and neurological disorders, including psychosis, acute stress, depression, schizophrenia, autism, and addiction (24–32). However, these studies are limited as 5hmC marks were examined either in a small subset of candidate genes (human studies) or in a genome-wide context using animal models, which may not recapitulate the epigenetic underpinnings of human disease. Recently, 5hmC genome-wide distribution was characterized for 19 human tissues derived from 10 organ systems (33). However, only limited studies are available exploring the genome-wide 5hmC profile in the human brain and its regulatory role in gene expression (34). One study by Madrid et al. performed genome-wide 5hmC profiling on human and non-human primates and demonstrated a mediatory role of 5hmC in neuronal-related processes associated with the evolution of the human brain (35). Two studies have shown decreased global DNA hydroxymethylation levels in the blood of patients with BD compared to control individuals (36, 37). However, since these studies were performed on peripheral blood, little was revealed about 5hmC levels in the brain among genes and pathways with pathologic relevance. By characterizing the epigenetic landscape, particularly 5hmC patterns, and the changes occurring during neurodevelopment, we can gain a better understanding of early-life, brain-related processes that may predispose for neuropsychiatric disease such as BD. Toward this end, we performed a pilot study to generate genome-wide 5hmC profiles from induced pluripotent stem cells (iPSCs) and neuronal stem cells (NSCs) derived from two individuals with BD and their unaffected siblings (*n* = 4). Our aim is to identify 5hmC changes in a two-stage, stem cell model for neurodevelopment to map differentially hydroxymethylated sites and wider regions, identify key genetic drivers involved in neuronal differentiation, and investigate whether such 5hmC changes are associated with BD risk.

## Materials and methods

### Subjects and sample collection

This study was conducted with approval from the Institutional Review Board (IRB) at the University of Texas Health Science Center at San Antonio. All procedures were performed in accordance with IRB guidelines and all samples and information were obtained with informed written consent. We recruited two adolescent patients (aged 9–18 years) with bipolar disorder (one male, one female) and their same-sex age-matched (±3 years) unaffected siblings; all participants were Hispanic. The affective module of the Washington University in St. Louis Kiddie-Schedule for Affective Disorders and Schizophrenia (WASH-U KSADS; kappa > 0.9 for diagnostic reliability) (38, 39) and the Kiddie-Schedule for Affective Disorders and Schizophrenia, Present and Lifetime (kappa 0.77–1.00 for diagnostic reliability) (40) were administered to parents and children in separate interviews by a board-certified psychiatrist to evaluate for the presence of current and lifetime psychiatric disorders. DSM-V criteria were used to determine current and lifetime psychiatric diagnoses. Blood was collected from each individual in Cell Preparation Tubes (CPT; BD Bioscience, Franklin Lakes, NJ, United States) for isolation of white blood cells, according to the manufacturers’ instructions. The white blood cell pellet was suspended in FBS containing 10% DMSO and stored in liquid nitrogen.

### Generation and maintenance of iPSCs and NSCs

White blood cells were sent to the Yale Stem Cell Center (New Haven, CT) for iPSC generation with Sendai virus mediated delivery of the four Yanamka reprogramming factors (Klf4, Oct, Sox2, and c-myc). iPSC clones were expanded to passage 10 (P10) to clear the non-integrating Sendai virus and underwent karyotyping by Cell Line Genetics (Madison, WI) to ensure normal karyotype. iPSCs were maintained in mTeSR1 media (Stem cell technologies, Cambridge, MA, United States) in six well plates coated with hESC-Qualified Matrigel (Corning, Durham, NC, United States). Media was changed every day and cells were sub-cultured twice a week or depending upon the size of the colonies using ReLeSR (Stem Cell Technologies).

Induction of NSCs from iPSCs was performed using PSC Neural Induction media (ThermoFisher Scientific, Waltham, MA, United States) as per the manufacturers’ recommendation with slight modification. Briefly, iPSCs were dissociated with ReLSR and transferred to a 15 mL tube for centrifugation at 200 *g* for 3 min. The pellet was resuspended in 1 mL of pre-warmed Accutase cell dissociation reagent (ThermoFisher Scientific) and incubated for 5 min at 37°C. Cells were dissociated to make a single cell suspension by vigorously pipetting up and down and then plated on Matrigel coated six well plates at a seeding density of 3 × 10^4^ cells/cm^2^ in mTeSR1 media. The following day, the media was replaced with PSC Neural Induction Medium with neural induction supplement (ThermoFisher Scientific) and maintained for additional 4 days of neural induction. Any non-neural differentiation was removed manually at day 4 using a Pasteur pipette. On day 7 when the cells reached maximum confluence, they were treated with Accutase, dislodged using a scraper and cell clumps were transferred to a 15 mL tube and broken up by pipette then passed through 100-μm strainer. Cells were centrifuged at 300 *g* for 4 min and washed once with PBS before resuspension in Neural Expansion Medium (ThermoFisher Scientific) with a final concentration of 5 μM of ROCK inhibitor Y27632 (Sigma-Aldrich, St. Louise, MO, United States) and then plated on Matrigel coated plates at a seeding density of 1 × 10^5^ cells/cm^2^. Media was replaced the next day with complete Neural expansion media without ROCK inhibitor. Cells reached confluence at day 6 and were further expanded and maintained in complete Neural Expansion Medium.

### Immuno-fluorescence microscopy

To confirm the purity of iPSCs and induction of NSCs (from iPSCs), cells were probed for typical iPSC/NSC markers using immunocytochemistry (Supplementary Figure 1). We used OCT4, Sox2, and Nanog antibodies for iPSCs and Sox2, Nestin and SSEA-4 for NSCs; details for all antibodies are provided in Supplementary Table 1. All antibodies were diluted in PBS containing 1% BSA and 0.1% sodium azide (Sigma-Aldrich, St. Louis, MO, United States). Cells were grown on sterile coverslips after coating with Matrigel and fixed with 2% paraformaldehyde in PBST (PBS with 0.1% Tween-20) for overnight incubation at 4°C. After fixation, cells were permeabilized with permeabilization buffer (PBS with 0.5% Saponin, 0.05% TritonX100; Sigma-Aldrich) for 10 min at 4°C then washed with cold wash buffer [PBST with 0.01% bovine serum albumin (BSA)] and blocked with 2% FBS before overnight incubation with primary antibodies at 4°C. After incubation with the primary antibodies, cells were washed and incubated for 1 h at room temperature in the dark with fluorescently labeled secondary antibodies. Cells were washed and mounted using mounting media containing DAPI (Vector Laboratories, Newark, CA, United States) and imaged at 10X magnification.

### Reduced representation hydroxymethylation profiling

Reduced representation hydroxymethylation profiling libraries were prepared from 100 ng DNA extracted from iPSC and NSC lines generated from two patients with BD and their unaffected siblings (*n* = 8 libraries). Libraries were generated using the RRHP 5-hmC Library Prep Kit (Zymo Research, Irvine, CA, United States), following manufacturer’s recommendations. Briefly, samples were digested with the *MspI* enzyme, which cuts DNA at CpG sites (providing 93% coverage of CpG islands), and then adapterized in a manner that recreates the *MspI* restriction site between the P5 adapter and genomic fragments. Samples underwent glucosylation, which modifies 5hmC positions at the adapter junction and protects them from further digestion. A second round of digestion with *MspI* cleaves the P5 adapter from all genomic fragments that are not glucosylated (i.e., non 5hmC sites) such that during subsequent PCR amplification, only genomic fragments containing 5hmC sites are amplified. Amplified cDNA libraries were quantified and assessed for quality using the Agilent TapeStation, then 10pM cDNA was sequenced on the Illumina HiSeq 2500. Libraries were multiplexed and sequenced in a single-read 50 bp run. PhiX DNA was spiked-in at 30% for each run to gain complexity of the library due to the degeneracy of the first several cycles.

### Data analysis

Sequences were de-multiplexed as *.fastq files and filtered for sequences harboring the P5 adapter “CCGG” at the 5′ end (i.e., representing a protected 5hmC site). Quality control metrics were obtained with the program FastQC v.0.11.5 (18), including quality scores per base, per flow cell tile, and per sequence. The P7 adapter “CG” and potential low-quality reads at the 3′ end were removed using Trimmomatic v.0.36 (19) [minimum length of 35 bp; 338,063 reads (0.5%) removed] and aligned to the hg19 human genome reference with bowtie 2 (20) using “very-fast” mode (alignment rates ranged from 97 to 98% for the different cell lines). Each of the 1,979,923 aligned 5hmC sites were annotated to gene locations (exon, UTR, 1-kb upstream promoter region) and CpG islands, including adjacent shores (2-kb) and shelves (2–4 kb), using hg38 liftover and GENCODE v.39 data available from the UCSC Genome Browser.

Read counts were tabulated and underwent quantile normalization using the R package preprocessCore (21) within the cell types. Sites with low 5hmC counts across samples were filtered out using the edgeR function *filterByExpr* (minimum 10 normalized counts each in a minimum of three iPSC or NSC samples) (41), leaving 124,603 CpG sites available for differential hydroxymethylation analysis. Count data were converted to log base 2 counts per million (CPM) using the VOOM transformation in the limma R package (22) by fitting a mean–variance trend (adjusted for age and sex). Paired *t*-tests were performed on the CPM values to identify differential hydroxymethylation among the filtered sites, providing a conservative and readily interpretable statistic. However, with small sample sizes, classical *t*-tests can lead to lower power and increased bias due to low variance (42). Hence, moderated *t*-tests were also computed with limma (22), which adjusts variances *via* the empirical Bayes method (i.e., borrowing information *across sites* for smoothing or shrinking variances) and increases degrees of freedom to improve power. Any power advantage, however, may be partially offset by higher false positive rates, especially for small sample sizes and homoscedastic differential expression (DE) approaches (42–44). For the VOOM-adjusted data in this study, the moderated *t*-test yielded negligible power gains [median |t| = 0.79 (*p* = 0.45); *f*(*p* < 0.05) = 0.021; Supplementary Figure 2] and an increase in false positives among top results. Given the implications for gene list composition for downstream enrichment testing of biological pathways and network construction, the more conservative, classical t-test results were examined in this study. Differentially hydroxymethylated regions (DhMR) were assessed using a sliding window approach (bin size of nine sequential 5hmC sites based on chromosome location; window size <50 kb) of Fisher’s combined probability for one-sided t-test *p* values.

Functional annotation and enrichment testing of genes harboring differentiated 5hmC loci were performed with the online tool DAVID v. 2021,^1^ targeting Gene Ontology (GO) terms and KEGG pathways. Protein–protein interaction (PPI) networks were constructed using STRING v. 11.5 (23), with a “high” confidence interaction score threshold (0.7) and using all available PPI sources. Two-way hierarchical clustering *via* UPGMA (unweighted pair group method with arithmetic mean) and heatmaps of Z-scores for VOOM-adjusted 5hmC levels for the top iPSC-NSC differentiated sites were generated in the R package “gplots” v. 3.5.3. Dendrograms were constructed using a straightforward Pearson correlation dissimilarity score for 5hmC levels, 1−*r*, evaluating the linear relationships between different iPSC and NSC cell lines and between the differentiated sites.

## Results

### Mapping of 5hmC sites to genomic features

**Figure 1:**
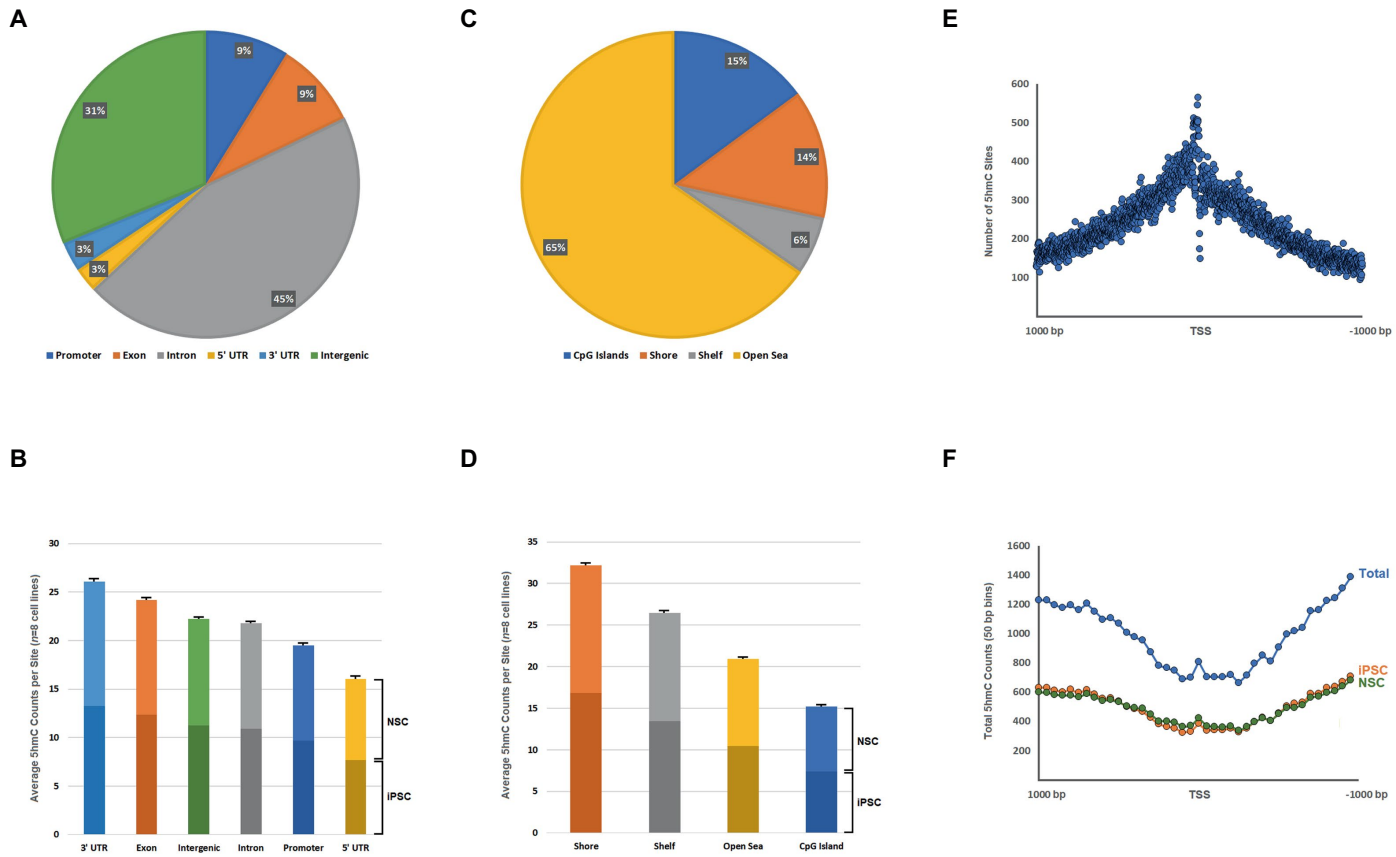
**(A)** Pie chart of the proportions of 5hmC sites (*n* = 1,979,923) mapped to the following genome features: exon, intron, 5′ UTR, 3′ UTR, promoter region (1-kb upstream of TSS), and intergenic; **(B)** Bar chart of mean normalized 5hmC counts per site for all eight cell lines (with SEM bars) according to different genome categories (iPSC-and NSC-specific means indicated by differential bar shades); **(C)** Pie chart of the proportions of 5hmC sites mapped to the following CpG-related genomic landscapes: CpG island (CGI), CpG shore (2 kb region adjacent CGIs), CpG shelf (2–4 kb from CGI boundaries), and open sea; **(D)** Bar chart of mean normalized 5hmC counts per site for all eight cell lines (with SEM bars) according to different CpG landscape types (iPSC-and NSC-specific means indicated by differential bar shades); **(E)** Distribution of the total number of observed 5hmC sites according to distance from the TSS (±1,000 bp) for all annotated genes; and **(F)** Distribution of total normalized 5hmC counts according to distance from the TSS (±1,000 bp; 50-bp bins) for all annotated genes.

We confirmed pluripotency and specificity of our cell lines by immunofluorescence, using standard markers for iPSCs (Oct4, Sox2, and Nanog; Supplementary Figure 1A) and NSCs (Sox2, Nestin, and SSEA-4; Supplementary Figure 1B). Following analysis of RRHP sequencing data, 1,979,923 5hmC sites were mapped, according to their genomic features—exon, intron, UTRs, 1-kb upstream promoter region and intergenic region (Figure 1A; Supplementary Table 2). Our combined analysis (of all iPSC and NSC lines) mapped 5hmC sites to introns (45%), intergenic regions (31%), exons (9%), promoters (9%), 5′ UTRs (3%) and 3′ UTRs (3%). When the mean 5hmC read counts (normalized) per site were calculated for all eight cell lines (both iPSC and NSC), we see that the highest mean 5hmC levels are found in the 3′ UTR (26 counts per site), followed by exon (24 counts per site), intergenic (22 counts per site), intron (22 counts per site), promoter (19 counts per site), and 5′ UTR (16 counts per site; Figure 1B, Supplementary Table 3). Analysis of 5hmC distribution according to CpG island proximity, localized 65.4% of 5hmC sites to open sea, 14.9% to CpG islands, 13.6% to CpG shores (2 kb from CGI), and 6.2% to CpG shelves (2–4 kb from CGI; Figure 1C and Supplementary Table 2). Mean 5hmC levels were highest in CpG shores (32 counts per site), followed by CpG shelves (26 counts per site), open sea (21 counts per site), and CpG islands (15 counts per site, Figure 1D, Supplementary Table 3). Moreover, we identified a relatively high density of 5hmC sites at transcription start sites (TSS) across all annotated genes, with the number of 5hmC sites decreasing linearly with increasing distance from the TSS (1,000 bp up/downstream; Figure 1E). Interestingly, the spatial pattern becomes inverted for normalized 5hmC counts summed for 50 bp bins around the TSS (Figure 1F), with lower 5hmC levels observed closer to the TSS, both for iPSCs and NSCs, despite the higher concentration of 5hmC sites.

### Comparison of 5hmC sites and DhMRs in iPSC-NSC pairs

**Figure 2:**
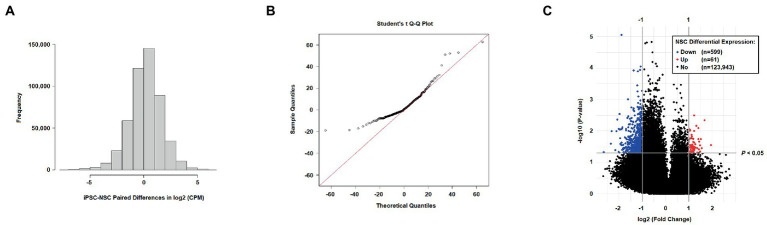
**(A)** Distribution of iPSC-NSC paired differences in log base 2 counts per million (CPM; VOOM transformation); **(B)** Student’s *t* quantile-quantile (Q-Q) plot for results from *t*-tests comparing VOOM-adjusted 5hmC counts between iPSC and NSC cell lines; and **(C)** Volcano plot of differential 5hmC expression (log base 2 FC; log base 10 *t*-test *p* values) among filtered sites for NSC cell lines relative to iPSC expression levels (downregulation defined as FC < −1 and *p* < 0.05; upregulation defined as FC > 1 and *p* < 0.05).

For comparative hydroxymethylation analysis, 124,603 CpG sites, filtered for low read counts, were assessed. The VOOM-adjusted CPM values showed normal distribution (Figure 2A), with the distribution of statistics from t-tests of iPSC-NSC pairs revealing deflation in the negative tail of a Q-Q plot (Figure 2B). This is a reflection of a significant skew in the 5hmC fold changes between iPSC and NSC cell lines (Figure 2C), with 53.8% of tested sites exhibiting negative FC (i.e., NSC *hypo*-hydroxymethylation), but increasing to 89.4% among nominally significant t-test results at *p* < 0.05 (*n* = 3,837; none are epigenome-wide significant). In the volcano plot (Figure 2C), differentiated 5hmC sites are defined as −1 ≥ FC ≥ 1 and *p* < 0.05, with 599 sites defined as down-regulated and only 61 as up-regulated, further underscoring the genomic reduction of 5hmC levels in NSCs. The 10 most differentially hydroxymethylated sites between iPSCs and NSCs (nominal significance) are shown in Table 1; all nominally significant associations (*p* < 0.05) are shown in Supplementary Table 4. Notably, all 10 of these nominally significant differentially hydroxymethylated sites exhibit reduced 5hmC levels in NSCs, and most are located within a transcription factor binding site (TFBS) or enhancer-like signature, as identified by ENCODE.

**Table 1:** Top-10 paired *t*-test results between iPSC and NSC cell lines for log2 (CPM) 5hmC.

Chr	Position (bp)*	Gene/lncRNA	Genomic Feature**	Log2 FC^λ^	*P*-value^#^
8	139,706,584		CpG Shore; TFBS (*KCNK9*)	−1.89	8.8 × 10^−06^
2	8,116,853	*LINC00298*	Intron	−0.60	1.5 × 10^−05^
6	26,104,997		CpG Shore; pELS; TFBS (*HIST1H4C*)	−0.81	1.6 × 10^−05^
11	118,534,285	*TMEM25*	Exon; CpG Shelf; dELS; TFBS (*TMEM25*)	−0.88	1.7 × 10^−05^
4	182,825,846		pELS; TFBS (*TENM3*)	−0.56	3.2 × 10^−05^
16	2,252,003	*ECI1*	Promoter (PLS); CpG Shore; TFBS (*ECI1*)	−0.57	6.4 × 10^−05^
1	125,569,926			−0.42	7.3 × 10^−05^
12	113,820,460	*RBM19*	Intron; dELS	−1.08	8.9 × 10^−05^
14	33,938,762	*EGLN3*	Intron; dELS; TFBS (*EGLN3*)	−0.91	1.1 × 10^−04^
12	124,544,085	*NCOR2*	Intron; CpG Shore; pELS	−1.09	1.1 × 10^−04^

Top-10 classic paired *t*-test results for VOOM-adjusted counts. Total of 124,603 5hmC sites were tested, filtered from the total data set using the edgeR function filterByExpr (minimum 10 normalized counts in a minimum of three iPSC or NSC samples).

* Reference assembly hg38.

** Annotations using UCSC Genome Browser (GRCh38.p13). Promoter-like signature (PLS), proximal enhancer-like signature (pELS), and distal enhancer-like signature (dELS) are based on *ENCODE Registry of Candidate cis-Regulatory Elements* (cCREs). Additionally, Transcription Factor Binding Sites (TFBS) based on regulatory regions curated by Open Regulatory Annotation (ORegAnno) (45), with the target gene provided in parentheses.

λ Negative log base-2 fold-change (log2 FC) represents reduced (i.e., hypo-) hydroxymethylation levels among NSC cell lines relative to iPSC cell lines. For nominally significant t-test results (*p* < 0.05; *n* = 3,837), 89.4% of FC estimates are negative. Overall, 53.8% of *t*-test results (*n* = 124,603) exhibit negative FCs.

# FDR > 0.51 (Benjamini & Hochberg method).

The DhMRs between iPSC and NSC cell lines were identified using a sliding window computation of Fisher’s combined probability test of one-sided *t*-test *p* values for reduced NSC 5hmC expression. The top-10 DhMRs are listed in Table 2, with most being found in enhancer-like signatures or CGI shores, underscoring their potential transcriptional function. The top DhMRs were found to be located in the genes neurofibromin 2 (*NF2*; *p* = 8.0 × 10^−05^), calcium voltage-gated channel subunit alpha1 H (*CACNA1H*; *p* = 1.6 × 10^−4^), and transmembrane protein 240 (*TMEM240*; *p* = 2.2 × 10^−04^).

**Table 2:** Top-10 differential hydroxymethylation regions (DhMRs) between iPSC and NSC cell lines.

Chr	Position (bp)*	Size (bp)	Gene/lncRNA	Regulatory Elements**	Fisher’s *p*-value^#^
22	29,668,338−29,704,694	36,357	*NF2*	CGI shore (3); dELS (7)	8.0 × 10^−05^
16	1,155,624−1,158,667	3,044	*CACNA1H*	CGI shore (9); pELS (5)	1.6 × 10^−04^
1	1,523,832–1,539,426	15,595	*ATAD3A*; *TMEM240*	CGI (2); CGI shore (6); dELS; pELS (2);	2.2 × 10^−04^
9	129,489,725−129,494,026	4,302	*LINC00963*	CGI shore (5); pELS (6); PLS (2)	2.5 × 10^−04^
9	126,531,343–126,534,992	3,650		dELS (3); pELS (2)	2.7 × 10^−04^
11	64,567,563–64,568,425	863	*SLC22A11*	CGI (6); CGI shore (3)	2.8 × 10^−04^
19	38,649,514–38,663,503	13,990	*ACTN4*	CGI shore (2); dELS (5); pELS	3.4 × 10^−04^
11	69,437,395–69,442,827	5,433	*LINC02952*	CGI shore (2); dELS (2)	4.3 × 10^−04^
1	3,350,517–3,355,176	4,660	*PRDM16*	dELS (3)	4.7 × 10^−04^
19	5,667,847–5,691,761	23,915	*HSD11B1L; RPL36*; *SAFB*	CGI (2); CGI shore (3); dELS (4); pELS (2)	5.0 × 10^−04^

DhMRs were identified using a sliding window computation of Fisher’s combined probability test of one-sided *t*-test *p* values for reduced NSC 5hmC expression (sliding window of nine sequential 5hmC sites based on chromosome location; window size <50 kb).

* Reference assembly hg38. Range of 5hmC sites comprising DhMR.

** Annotation for regulatory elements *per site* using UCSC Genome Browser (GRCh38.p13). PLS, promoter-like signature; pELS, proximal enhancer-like signature; and dELS, distal enhancer-like signature as identified by *ENCODE Registry of candidate cis-Regulatory Elements* (cCREs); and CGI is CpG island. Multiple annotations of a single type indicated in parentheses. CGI shore is defined as a site within 2 kb a CGI.

# Not epigenome-wide significant after Bonferroni correction based on 13,845 non-overlapping bins.

### Functional annotation of differentiated 5hmC sites in iPSC-NSC pairs

**Figure 3:**
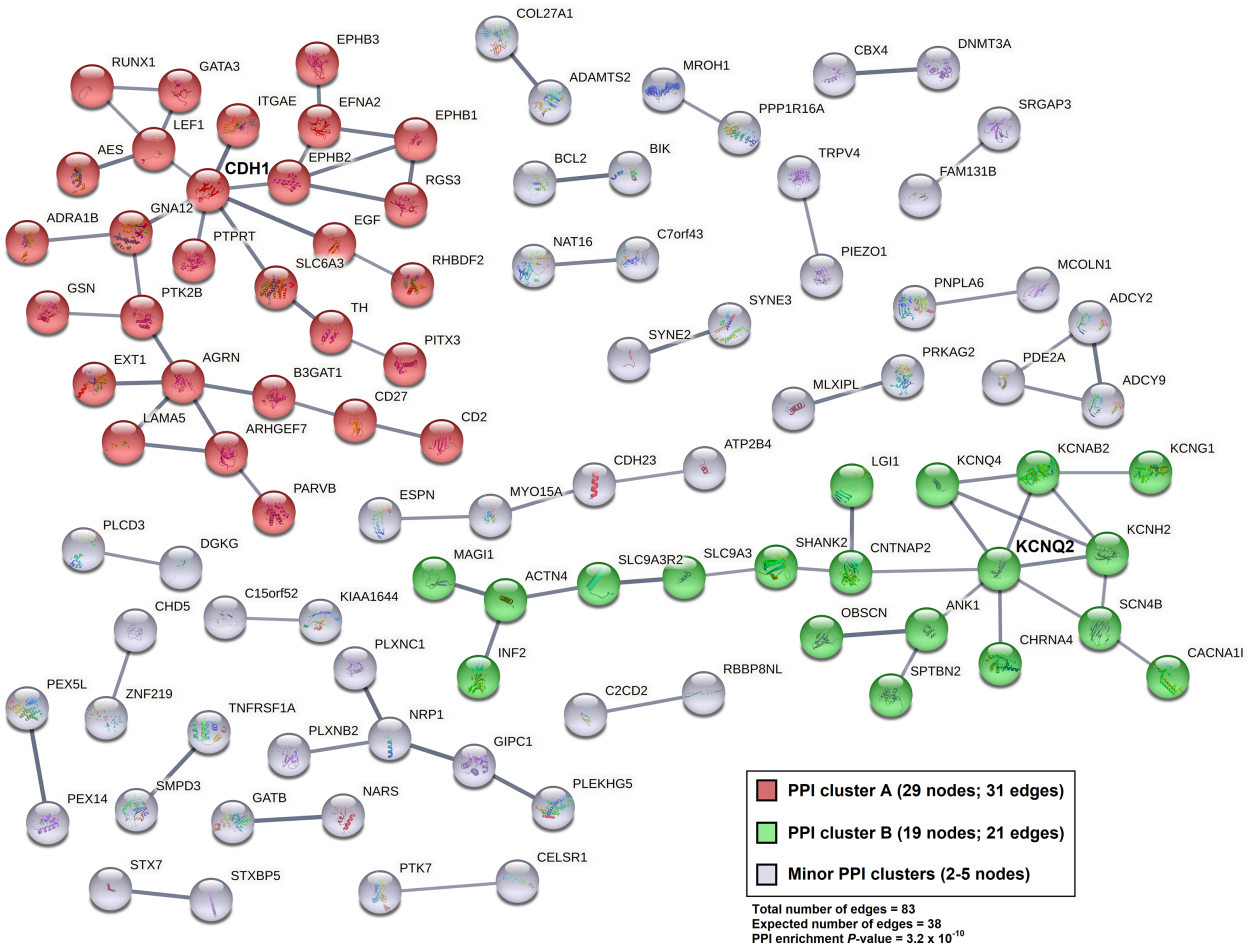
Protein–Protein Interaction (PPI) network (STRING v. 11.5; confidence interaction score > 0.7) for genes harboring sites with reduced 5hmC expression levels (*p* < 0.05; 3,432 sites localized to 1,627 genes) among NSC cell lines relative to their iPSC pairs.

Gene set enrichment analysis was performed for Gene ontology (GO) term clustering [for Biological Process (BP), Cellular Component (CC), and Molecular Function (MF)] for 3,432 differentiated 5hmC sites, localized to 1,627 genes, with reduced NSC expression (*p* < 0.05). The 10 most enriched functional annotations are listed in Table 3 (observed genes in each functional annotation category are listed in Supplementary Table 5). The top enrichment is for genes and their products localized to the plasma membrane component of the cell (28.1% of the gene list fall in this category; FDR = 9.1 × 10^−12^), followed by perinuclear region of cytoplasm (5.8% of gene list; FDR = 2.0 × 10^−6^). Significant enrichment of genes related to axon guidance (2.3% of gene list; FDR = 2.1 × 10^−6^), neuron projection (3.5% of gene list; FDR = 2.4 × 10^−6^) and neuronal cell body (3.5% of gene list; FDR = 2.0 × 10^−5^) was also noted. The 1,627 genes showing decreased 5hmC levels in NSCs were further analyzed by creating protein–protein interaction (PPI) networks (confidence interaction score > 0.7; Figure 3). In total, 83 PPI edges were observed, significantly greater than the expected 38 edges (*p* = 3.2 × 10^−10^), indicating significant PPI connectivity among genes harboring reduced 5hmC levels in NSCs. Two major clusters were observed: “A” (red), with 29 gene nodes (*CDH1* is a hub gene); and “B” (green), with 19 gene nodes (*KCNQ2* is a hub gene).

**Table 3:** Top-10 enriched functional annotations for differentiated 5hmC sites (nominal *p* < 0.05) with reduced NSC expression (3,432 sites localized to 1,627 genes).

Category	Term	Count	Percent (%)	FDR
GO Term (CC)	GO:0005886 “plasma membrane”	458	28.1	9.1 × 10^−12^
GO Term (CC)	GO:0048471 “perinuclear region of cytoplasm”	94	5.8	2.0 × 10^−06^
GO Term (CC)	GO:0005925 “focal adhesion”	62	3.8	2.0 × 10^−06^
KEGG Pathway	hsa04360 “Axon guidance”	38	2.3	2.1 × 10^−06^
GO Term (CC)	GO:0043005 “neuron projection”	57	3.5	2.4 × 10^−06^
GO Term (MF)	GO:0003779 “actin binding”	55	3.4	1.7 × 10^−05^
GO Term (CC)	GO:0070161 “anchoring junction”	64	3.9	2.0 × 10^−05^
GO Term (CC)	GO:0043025 “neuronal cell body”	57	3.5	2.0 × 10^−05^
GO Term (MF)	GO:0051015 “actin filament binding”	41	2.5	3.3 × 10^−05^
GO Term (CC)	GO:0098685 “Schaffer collateral—CA1 synapse”	21	1.3	7.7 × 10^−05^

Gene set enrichment analysis was performed using the online tool DAVID (https://david.ncifcrf.gov), targeting Gene Ontology (GO) terms [Biological Process (BP), Cellular Component (CC), and Molecular Function (MF)] and KEGG pathways.

### Two-way hierarchical clustering of iPSC-NSC data

**Figure 4:**
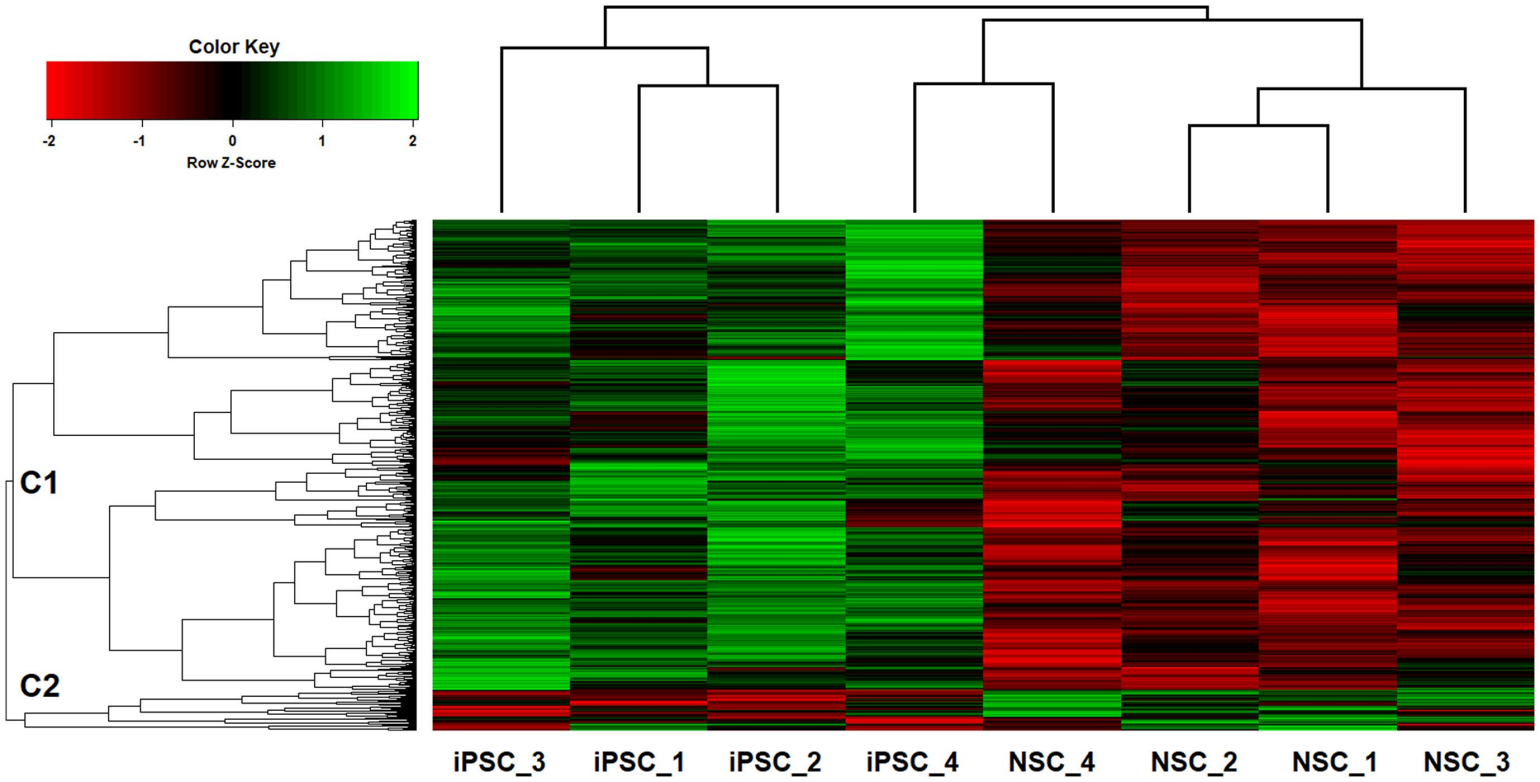
Two-way UPGMA clustering of VOOM-adjusted 5hmC expression levels for top iPSC-NSC differentiated sites (*p* < 0.01; *n* = 701), with aligned heatmap of *Z*-scores (columns represent different iPSC and NSC cell lines; rows represent genome-wide sites).

Two-way hierarchical clustering was performed to examine the broader co-expression patterns among the top iPSC-NSC differentiated sites (*p* < 0.01; *n* = 701; Figure 4). For the dendrogram in the top-margin, iPSCs and NSCs cluster separately, except for the iPSC and NSC pairing for sample #4, reflecting a linear relationship between the iPSC and NSC 5hmC data, despite conspicuous differences in overall 5hmC levels at the various sites. The second dendrogram, in the left-margin, reveals two primary branches among the 5hmC sites, C1 and C2, with C2 representing a smaller, outlier group (*n* = 56). The two dendrograms are aligned as columns and rows in the heatmap, respectively, and intersect within a color-coded matrix of Z-scores for adjusted 5hmC levels across the cell lines and differentiated sites. Among the NSCs and for sites comprising the major C1 cluster, reduced 5hmC levels are observed, underscoring the mostly unidirectional changes in 5hmC levels.

### Comparison of 5hmC levels between bipolar disorder cases and their unaffected siblings

We assessed 5hmC levels in NSCs derived from BD cases and their unaffected sibs (*n* = 4), observing a total of 3,244 nominally significant (*p* < 0.05) differentially hydroxymethylated sites, although none of these reached genome-wide significance (Supplementary Table 6, Supplementary Figure 3). Table 4 shows the most differentially hydroxymethylated sites between BD cases and their unaffected siblings. The top differentiated site is within the exonic region of the lncRNA ENSG00000253235, and is associated with a proximal enhancer-like signature (pELS) and TFBS. The next top result is for a site within the gene Cut Like Homeobox 2 (*CUX2*), a transcription factor involved in the control of neuronal proliferation and differentiation in the brain (46). In addition to analysis of single sites, we performed analysis of differentially hydroxymethylated regions, using a sliding window approach. The top 10 DhMRs are shown in Table 5, with the top DhMR in the gene *DLGAP4*, encoding a membrane-associated guanylate kinase found at the postsynaptic density in neuronal cells. We also examined 5hmC sites within known BD risk genes, identified from a recent GWAS meta-analysis (*n* = 64) (47). Within these BD risk genes, we identified 11 5hmC sites that showed nominally significant (*p* < 0.05) differential hydroxymethylation between BD cases and unaffected siblings; most of these sites were within intronic regions (Supplementary Table 7). The top identified differentially hydroxymethylated sites located within BD risk genes were found in Erb-B2 Receptor Tyrosine Kinase 2 (*ERBB2*), Glutamate Ionotropic Receptor NMDA Type Subunit 2A (*GRIN2A*), B-cell lymphoma/leukemia 11B (*BCL11B*), plectin (*PLEC*), and SH3 and Multiple Ankyrin Repeat Domains 2 (*SHANK2*).

**Table 4:** Top-10 differentiated sites for 5hmC levels in BD case and sib pairs in NSC cell lines.

Chr	Position (bp)*	Gene/lncRNA	Genomic Feature**	Log2 FC^λ^	*P*-value^#^
8	73,370,576	ENSG00000253235	Exon; pELS; TFBS	−0.85	1.4 × 10^−05^
12	111,293,515	*CUX2*	Exon	1.88	2.4 × 10^−05^
7	128,168,609		CpG Island; TFBS (miRNA 129–1)	−1.76	2.9 × 10^−05^
22	37,911,350	*MICALL1*	Intron	0.46	3.1 × 10^−05^
4	3,462,651	*DOK7*	Promoter; CpG Shore; pELS; TFBS (*DOK7*)	−1.43	3.6 × 10^−05^
6	37,539,622		dELS	0.57	3.7 × 10^−05^
20	62,980,185		CpG Shore; dELS	0.86	3.8 × 10^−05^
19	50,661,877	*SHANK1*	Exon; CpG Shore; dELS	0.92	5.6 × 10^−05^
1	234,206,319		dELS	−1.76	6.4 × 10^−05^
18	48,547,816	*CTIF*	Intron	−0.78	6.8 × 10^−05^

Top-10 paired *t*-test results for VOOM-adjusted counts from NSC cell lines. Total of 101,673 of the 124,603 5hmC sites were tested, filtered using the edgeR function *filterByExpr* (minimum 10 normalized counts in two of the four samples).

* Reference assembly hg38.

** Annotations using UCSC Genome Browser (GRCh38.p13). Promoter-like signature (PLS), proximal enhancer-like signature (pELS), and distal enhancer-like signature (dELS) are based on *ENCODE Registry of Candidate cis-Regulatory Elements* (cCREs). Additionally, Transcription Factor Binding Sites (TFBS) based on regulatory regions curated by Open Regulatory Annotation (ORegAnno), with the target gene provided in parentheses.

λ Positive log base-2 fold-change (log2 FC) represents lower (i.e., hypo-) hydroxymethylation levels among BD cases relative to unaffected sibs. For nominally significant *t*-test results (*p* < 0.05; *n* = 3,244), 57.2% of FC estimates are negative. Overall, 50.7% of *t*-test results (*n* = 101,673) exhibit negative FCs.

# FDR > 0.69 (Benjamini & Hochberg method).

**Table 5:** Top-10 differential hydroxymethylation regions (DhMRs) between BD cases and sibs for NSC cell lines.

Chr	Position (bp)*	Size (bp)	Gene/lncRNA	Regulatory Elements**	Effect in BD Cases	Fisher’s *p*-value^#^
20	36,307,561–36,320,198	12,638	*DLGAP4*	CGI shore (6); dELS (2); pELS (2)	↓ 5hmC	3.1 × 10^−03^
5	173,298,761–173,324,407	25,647	*STC2*	dELS (5)	↓ 5hmC	3.7 × 10^−03^
22	23,846,922–23,849,954	3,033		CGI (5); CGI Shore (3); dELS (4)	↑ 5hmC	5.7 × 10^−03^
4	2,266,369–2,285,757	19,389	*ZFYVE28*	CGI Shore (2); dELS (3)	↓ 5hmC	9.8 × 10^−03^
11	64,154,502–64,165,286	10,785	*MACROD1*	CGI Shore (7); dELS (3); pELS (6)	↑ 5hmC	1.1 × 10^−02^
7	26,620,615–26,628,209	7,595		dELS (5)	↓ 5hmC	1.1 × 10^−02^
12	3,033,058–3,055,770	22,713	*TEAD4*	dELS (6)	↓ 5hmC	1.2 × 10^−02^
11	69,862,436–69,862,865	430		dELS (7)	↓ 5hmC	1.4 × 10^−02^
16	630,468–631,341	874	*WFIKKN1*	CGI Shore (5); pELS (8)	↓ 5hmC	1.7 × 10^−02^
21	43,383,236–43,383,498	263			↓ 5hmC	1.9 × 10^−02^

DhMRs were identified using a sliding window computation of Fisher’s combined probability test of one-sided t-test *p*-values for NSC 5hmC expression among BD cases and unaffected sibs (sliding window of nine sequential 5hmC sites based on chromosome location; window size <50 kb).

* Reference assembly hg38. Range of 5hmC sites comprising DhMR.

** Annotation for regulatory elements *per site* using UCSC Genome Browser (GRCh38.p13). Abbreviations: promoter-like signature (PLS), proximal enhancer-like signature (pELS), and distal enhancer-like signature (dELS), as identified by *ENCODE Registry of candidate cis-Regulatory Elements* (cCREs); and CGI is CpG island. Multiple annotations of a single type indicated in parentheses. CGI shore is defined as a site within 2 kb a CGI.

# Not epigenome-wide significant after Bonferroni correction based on 11,297 non-overlapping bins.

The 10 most enriched functional annotations for genes containing differentially hydroxymethylated sites between BD cases and unaffected sibs in NSCs (*p* < 0.05) are listed in Table 6 (observed genes in each functional annotation category are listed in Supplementary Table 8). The top enrichment is for genes involved in the extracellular matrix component of the cell (FDR = 1.0 × 10^−8^), with other enrichments observed for the plasma membrane (FDR = 5.5 × 10^−5^), actin cytoskeleton (FDR = 3.1 × 10^−4^), and GTPase activator activity (FDR = 3.2 × 10^−3^).

**Table 6:** Top-10 enriched functional annotations for 5hmC sites differentiated between BD case and sib pairs for NSC cell lines (*p* < 0.05; localized to 1,582 genes).

Category	Term	Count*	Percent (%)	FDR
GO Term (CC)	GO:0031012 “extracellular matrix”	49	3.1	1.0 × 10^−08^
GO Term (CC)	GO:0005886 “plasma membrane”	400	25.3	5.5 × 10^−05^
GO Term (CC)	GO:0070161 “anchoring junction”	62	3.9	5.5 × 10^−05^
GO Term (CC)	GO:0005604 “basement membrane”	22	1.4	5.5 × 10^−05^
GO Term (CC)	GO:0015629 “actin cytoskeleton”	38	2.4	3.1 × 10^−04^
GO Term (CC)	GO:0044305 “calyx of Held”	10	0.6	3.1 × 10^−04^
GO Term (CC)	GO:0005856 “cytoskeleton”	62	3.9	7.7 × 10^−04^
GO Term (MF)	GO:0005085 “guanyl-nucleotide exchange factor activity”	38	2.4	1.2 × 10^−03^
GO Term (MF)	GO:0005201 “extracellular matrix structural constituent”	26	1.6	3.0 × 10^−03^
GO Term (MF)	GO:0005096 “GTPase activator activity”	41	2.6	3.2 × 10^−03^

Gene set enrichment analysis was performed using the online tool DAVID (https://david.ncifcrf.gov), targeting Gene Ontology (GO) terms [Biological Process (BP), Cellular Component (CC), and Molecular Function (MF)], and KEGG pathways.

* Bipolar disorder (BD) risk genes from the third GWAS meta-analysis of the PGC Bipolar Disorder Working Group (2021) (48) were observed among the top GO term enrichments: *CACNA1C*, *ERBB2*, *GRIN2A*, *PLEC*, *PLXNA4*, *RTN4RL1*, and *SHANK2*. All seven are part of the “plasma membrane” category.

## Discussion

Embryonic stem cells (ESCs) and iPSCs are known to exhibit high 5hmC levels, which are diminished upon differentiation, except in mature neuronal cells (20). Further, 5hmC profiles are highly dynamic during fetal brain development, with the highest levels of 5hmC observed in the mammalian adult brain (21, 49–53). Regional (23) and cell-specific (22) patterns of differential 5hmC are evident within the brain, suggesting that 5hmC may not just be important for neurodevelopment, but also for brain-related disease pathology. We therefore performed a pilot study to investigate the changes in 5hmC levels during differentiation of iPSCs to NSCs and examine whether there are differences in 5hmC profiles in NSCs of patients with bipolar disorder versus their unaffected siblings. Our combined analysis mapped 5hmC sites primarily to introns (45%) and intergenic regions (31%), with a smaller number of sites being mapped to exons (9%), promoters (9%), 5′ UTRs (3%) and 3′ UTRs (3%); these findings are concordant with those previously reported (54, 55). Interestingly, we observed high enrichment of 5hmC sites around transcription start sites, although 5hmC levels were lower at these sites around the TSS and increased at sites more distal (up to 1 kb) to the TSS. This is concordant with previous studies in human and mouse samples, that show enrichment of 5hmC levels distal to the TSS (up to ~1 kb) in genes with medium to high expression, but higher 5hmC levels around the TSS for genes with low or no expression (52, 56, 57). Poorly transcribed or untranscribed genes generally possess a peak of 5hmC at the TSS itself (58), thus 5hmC enrichment in this area may have an inhibitory effect. As for proximity to CpG islands, most 5hmC sites are open sea (65.4%), with the remainder mapped to CpG islands (14.9%), shores (13.6%), and shelves (6.2%), which is also consistent with previous reports (59, 60).

5-hydroxymethylcytosine is highly expressed in human brain relative to other tissues and is considered a stable epigenetic regulator of gene expression in neuronal development and differentiation (57, 61). Mechanistically, 5hmC is an intermediate of the demethylation process initiated by Tet1, it interferes with the maintenance of DNA methylation pattern by inhibiting the recruitment of DNMT1 (62, 63), however, its role as a stable epigenetic modification following reprogramming is becoming increasingly well-recognized. Studies in mouse ES cells show enrichment of 5hmC in intragenic regions, particularly at the 3′ end of actively transcribed Tet1 bound genes, as well as at the promoter regions of Tet1/PRC2-cobound (repressed) developmental regulators, suggesting that 5hmC enrichment may contribute to maintenance of both transcriptionally active and inactive chromatin states by functionally interacting with distinct histone modifications (60). In mouse brain tissues, 5hmC enrichment in the gene bodies of expressed genes is associated with increased gene expression, while repressed genes exhibited a marginal decrease in 5-hmC levels in rat brain (64, 65). However, several housekeeping genes with high expression have low or no 5hmC enrichment, suggesting a different regulatory mechanism (66). In *in vitro* models, the majority of 5hmC peaks in ESCs are shown to be lost in neural precursor/progenitor cells (NPCs), with some *de novo* DNA hydroxymethylation occurring at gene loci associated with mature neuronal functions (55). In addition to these distinct distribution differences, global 5hmC levels within NPCs is much lower than that of ESCs (55), a finding which we also confirmed in our analyses. This suggests that, at least in the early stages of neuronal differentiation (iPSCs → NSCs), a genomic reduction of 5hmC may be required. Moreover, upon differentiation of ESCs to NPCs, a prior study has shown dramatic changes in 5hmC levels in promoters, exons, and enhancers (67). However, higher levels of 5hmC have been reported in brain regions with neuronal function than in stem cell rich areas of the brain (49), suggesting there are likely increased levels of 5hmC in genes involved in neuronal differentiation and maturation. Also, acquisition of 5hmC during embryonic brain development has been reported (68, 69), demonstrating dynamic changes in 5hmC signatures throughout neuronal development.

In our single site analyses of iPSC and NSC 5hmC expression levels, although none of the differences were epigenome-wide significant, a number of interesting results were observed. Our most significant difference (*p* = 8.8 × 10^−6^) was observed for a TFBS for the target gene *KCNK9*, a potassium channel protein highly expressed in the cerebellum and involved in neuronal activity and migration (70). We also identified nominally significant differential hydroxymethylation among CpG sites within several other TFBS for target genes implicated in neuronal activity. These include *TMEM25* (*p* = 1.7 × 10^−5^), known to modulate neuronal excitability and potentially attenuate epileptic seizure-related behavioral phenotypes (71); *TENM3* (*p* = 3.2 × 10^−5^), a transmembrane protein associated with most excitatory synapses of the hippocampus and which likely promotes establishment and shaping of synaptic connections (48, 72); and *EGLN3* (*p* = 1.1 × 10^−4^), which has been linked to neuronal apoptosis (73). A site within the intron of *NCOR2*, which regulates gene expression by activating histone deacetylase 3, and whose loss is associated with memory impairment (74), also showed nominally significant differential hydroxymethylation (*p* = 1.1 × 10^−4^). Furthermore, the analysis of DhMRs for reduced NSC 5hmC expression identified the gene *NF2* as the top DhMR, which is involved in cytoskeletal dynamics and regulation of ion transport and is linked to peripheral nerve damage (75, 76). The second top DhMR was found in *CACNA1H*, which encodes a protein in a voltage-dependent calcium channel complex that has been implicated in epilepsy (77, 78). We also noted a DhMR in the *ACTN4* gene locus, which regulates neuronal structural plasticity (79) and observed altered hydroxymethylation in a distal enhancer-like signature of the *PRDM16* gene, which is a transcriptional regulator required for stem cell function in multiple neonatal tissues, including nervous system (80–82). Prdm16 acts as a chromatin modifying enzyme, shown to function as a histone 3 lysine 9 (H3K9) and histone 3 lysine 4 (H3K4) mono-methyltransferase (83), which also forms complexes with transcriptional co-factors and other histone modifying proteins to regulate gene expression (84). Prdm16 is well known for its role to control embryonic and postnatal neural stem cell maintenance and differentiation in the brain by activating a stage specific gene expression program to establish the organization of the cortex and by regulating the activity of transcriptional enhancers to repress genes that control migration of upper layer neurons (81, 82, 85).

Our functional annotation using GO analysis of nominally significant hypo-hydroxymethylated sites in NSCs (*p* < 0.05) indicated enrichment of genes associated with plasma membrane and for various neuronal functions, particularly those related to neuronal differentiation and migration (axon guidance, neuron projection, and neuronal cell body). The PPI network analysis of these hypo-hydroxymethylated sites in NSCs identified two major clusters, centering around the *CDH1* and *KCNQ2* gene hubs. Cdh1 is suggested to prevent the accumulation of cyclin B1 in terminally differentiated neurons thereby inhibiting neurons to enter the S phase of cell cycle, and is also associated with survival of postmitotic neurons (86). *KCNQ2*, belongs to the potassium channel gene family, and is known to regulate and maintain normal brain function (87, 88). Within the first cluster, centered around *CDH1*, we also identified other important genes involved in axon guidance, (*EPHB* family genes, *EFNA2*, *EXT1*, *GATA3*, and *LAMA5*) (89, 90) and within the second cluster, centered around *KCNQ2*, we identified genes involved in ion transmembrane transport (*KCNQ4*, *KCNAB2* and *KCNG1*) (91).

To explore potential 5hmC changes contributing to BD pathology during early neuronal differentiation, we analyzed 5hmC levels between BD cases and their unaffected sibs (*n* = 4) in NSCs. Again, given the very limited sample size, epigenome-wide significant loci were not observed. The most differentiated site between the BD and unaffected sibs is located at overlapping pELS and TFBS elements within the lncRNA ENSG00000253235, suggesting a regulatory role. Other potential risk sites among our top results with brain-related relevance include ones within *CUX2*, a transcription factor that controls neuronal proliferation, dendrite branching and synapse formation and is required for normal dendrite development of layer II-III neurons (92, 93); and DOK-7, an adaptor protein known to regulate neuromuscular synapse formation (94). Analyses of DhMR in BD cases also identified several potential risk genes with known functional roles in neuronal cells and brain development. These include *DLGAP4*, which encodes a membrane-associated guanylate kinase found at the postsynaptic density in neuronal cells and that plays a vital role in synaptic scaling by regulating the turnover rate of ionotropic and metabotropic glutamate receptors (95); *STC2*, which shows a neuroprotective effect during cerebral ischemia (96); and *TEAD4*, which belongs to a family of proteins known to regulate cortical development (97). To determine if there may be a role for 5hmC-related epigenetic regulation of known BD risk genes, we searched for differentially hydroxymethylated sites in 64 previously identified BD risk genes (47), identifying 11 sites with nominal significance (*p* < 0.05). The top result is for an intronic site in the gene *ERBB2*, which regulates the formation of neuromuscular synapse and muscle spindles (98). We also identified differentially hydroxymethylated sites in the promoter and distal enhancer-like signature (dELS) regions of *GRIN2A*, an important regulator of glutamate signaling that has been well associated with bipolar disorder (99).

It is important to acknowledge the limitations of this study, in particular the small sample size, which can lead to lower power and increased bias due to low variance. Moreover, we are also aware that due to the small sample size, the results might not be representative of all patients with bipolar disorder. As such, the results presented here are not necessarily informative of neurodevelopment in patients with bipolar disorder nor represent specific targets for clinical treatments. Rather, this pilot study serves as a benchmark for additional studies, demonstrating preliminary evidence of a role for hydroxymethylation changes during neurodifferentiation in patients with bipolar disorder. We utilized RRHP to identify 5hmC levels genome-wide, which is different to most published studies that typically use antibody-based immunoprecipitation. However, our findings related to the mapping of 5hmC sites and the general pattern of hypo-hydroxymethylation in NSCs compared to iPSCs was concordant with previously published studies, lending validity to our approach. Although we identified several genes with suggestive differential 5hmC signatures in iPSCs versus NSCs, and in BD cases versus unaffected siblings, results should be interpreted with caution, and validated with larger sample sizes. Our sib-pair design needs to be considerate of the higher genetic risk of bipolar disorder in unaffected siblings. Our iPSC-NSC analysis of these sib-pairs may reveal differential hydroxymethylation of genes specific to bipolar disorder (or its risk). However, since our iPSC-NSC results are highly concordant with prior studies, we are confident that both our experimental approach (RRHP) and samples used provide additional support for the role of differential hydroxymethylation in genes involved in neuronal differentiation and migration, plasticity, and synaptic connections. This is further highlighted by functional annotation, also implicating categories related to neuronal differentiation and migration. In our comparison of NSCs derived from patients with BD and their unaffected siblings, we identified differential hydroxymethylation in genes implicated in neuronal proliferation, cortical development and synaptic scaling, which may suggest a role for dysfunction of these in BD.

Our study has not identified potential regulatory functions of these differential 5hmC signatures, and prior studies indicate that the relationship between 5hmC and gene expression is complex, and dependent upon genic location and even gene function (60, 64, 100). Thus, more comprehensive studies targeting combined 5hmC and gene expression analyses to evaluate 5hmC regulatory function is warranted, and these will likely provide additional support for the importance of such epigenetic factors in neuronal differentiation. While our study focuses on understanding the pathophysiology of bipolar disorder, the implications of such findings could extend into clinical practice. First, the identification of differential 5hmC marks could serve as potential biomarker to identify the individuals who are at higher risk of developing BD, which can allow for better management of disease. Second, if 5hmC changes associated with BD show reversion upon treatment with common BD drugs, or alternate drugs that could be repurposed, these can also be used as a biomarker of disease prognosis. More comprehensive analyses, using larger sample sizes are necessary for validating the genes and pathways identified in our study, and expanded studies investigating the effect of therapeutic agents on 5hmC signatures in BD may further advance clinical applications.

## Data availability statement

The datasets for this article are not publicly available due to concerns regarding participant/patient anonymity. Requests to access the datasets should be directed to the corresponding author.

## Ethics statement

The studies involving human participants were reviewed and approved by Institutional Review Board (IRB) at the University of Texas Health Science Center at San Antonio. Written informed consent to participate in this study was provided by the participants’ legal guardian/next of kin.

## Author contributions

MC, AK, and MK designed the study and wrote the manuscript. DR and MC recruited patients and collected samples for use in this study. AK performed experiments. MK performed data analysis. All authors contributed to the article and approved the submitted version.

## Funding

This study was conducted with funding support from Texas Biomedical Research Institute in facilities constructed with support from Research Facilities Improvement Program Grant Numbers C06 RR013556 and C06 RR017515 from the National Center for Research Resources, National Institutes of Health. AK was supported by a Cowles Fellowship administered through Texas Biomedical Research Institute.

## Conflict of interest

The authors declare that the research was conducted in the absence of any commercial or financial relationships that could be construed as a potential conflict of interest.

## Publisher’s note

All claims expressed in this article are solely those of the authors and do not necessarily represent those of their affiliated organizations, or those of the publisher, the editors and the reviewers. Any product that may be evaluated in this article, or claim that may be made by its manufacturer, is not guaranteed or endorsed by the publisher.

## Footnotes

^1^ https://david.ncifcrf.gov

## Supplementary material

SUPPLEMENTARY FIGURE 1 Fluorescence microscopy images of **(a)** iPSCs and **(b)** NSCs demonstrating pluripotency (iPSC markers Oct4, Sox2 and NANOG; NSC marker Sox2) and early neuronal lineage (NSC markers Nestin and SSEA-4).

SUPPLEMENTARY FIGURE 2 Scatterplot of test scores generated from classical paired *t*-tests and moderated *t*-tests (limma) that compared iPSC and NSC 5hmC expression levels.

SUPPLEMENTARY FIGURE 3 **(a)** Student’s *t* quantile-quantile (Q-Q) plot for results from *t*-tests comparing VOOM-adjusted 5hmC counts NSC cell lines between BD cases and unaffected sibs; and **(b)** volcano plot of differential 5hmC expression (log base 2 FC; log base 10 *t*-test *P*-values) between BD cases and unaffected sibs for NSC cell lines (downregulation defined as FC < -1 and *P* < 0.05; upregulation defined as FC > 1 and *P* < 0.05).
