# Supplementary figures and images for: A pilot investigation of differential hydroxymethylation levels in patient-derived neural stem cells implicates altered cortical development in bipolar disorder
Source: Front Psychiatry. 2023 Apr 17;14:1077415. doi: 10.3389/fpsyt.2023.1077415 (PMC10150707; doi:10.3389/fpsyt.2023.1077415)

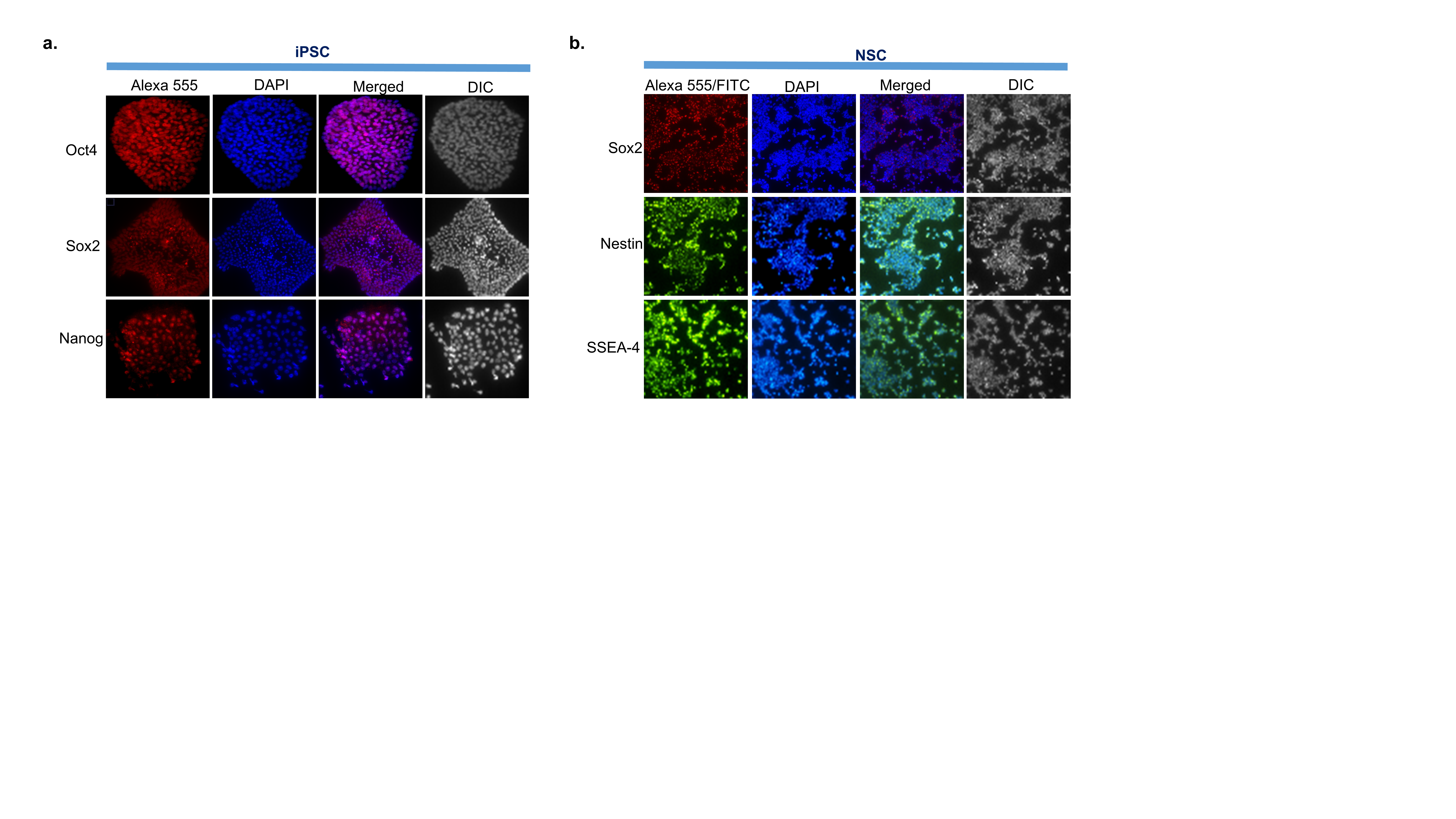

Supplement: SUPPLEMENTARY FIGURE 1 — Fluorescence microscopy images of (a) iPSCs and (b) NSCs demonstrating pluripotency (iPSC markers Oct4, Sox2 and NANOG; NSC marker Sox2) and early neuronal lineage (NSC markers Nestin and SSEA-4). [file Image_1.TIF]

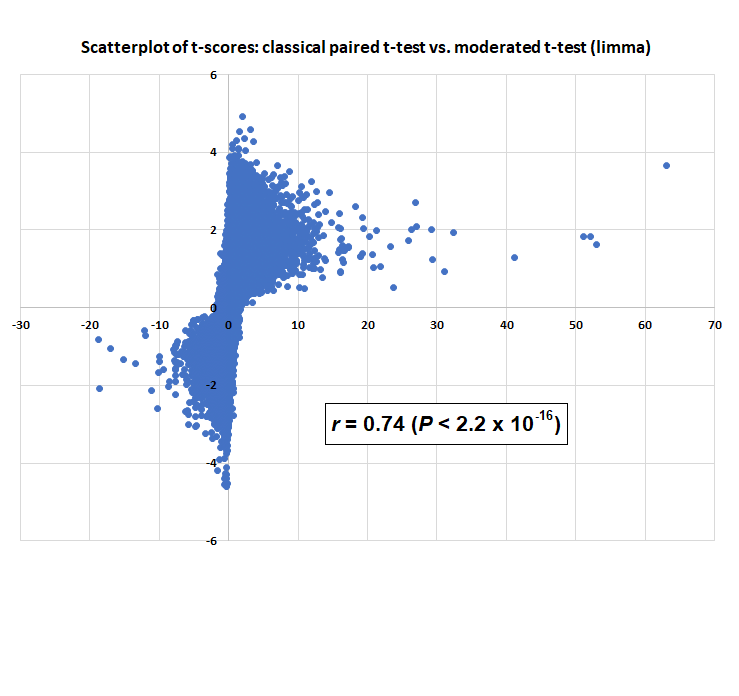

Supplement: SUPPLEMENTARY FIGURE 2 — Scatterplot of test scores generated from classical paired t-tests and moderated t-tests (limma) that compared iPSC and NSC 5hmC expression levels. [file Image_2.TIFF]

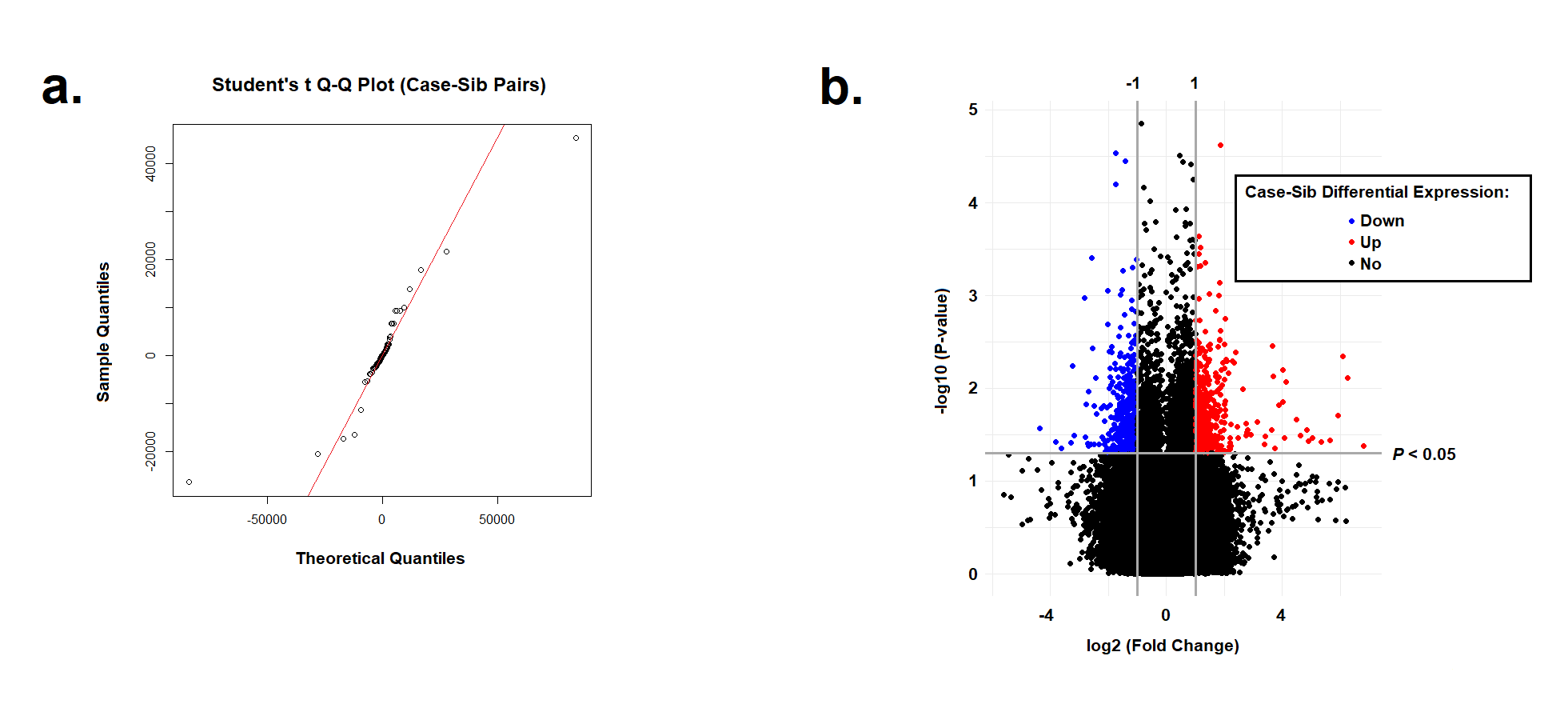

Supplement: SUPPLEMENTARY FIGURE 3 — (a) Student’s t quantile-quantile (Q-Q) plot for results from t-tests comparing VOOM-adjusted 5hmC counts NSC cell lines between BD cases and unaffected sibs; and (b) volcano plot of differential 5hmC expression (log base 2 FC; log base 10 t-test P-values) between BD cases and unaffected sibs for NSC cell lines (downregulation defined as FC < -1 and P < 0.05; upregulation defined as FC > 1 and P < 0.05). [file Image_3.TIFF]
